# Implementing the New Child and Adult Care Food Program’s Nutrition Standards in Boston

**DOI:** 10.5888/pcd17.190426

**Published:** 2020-06-18

**Authors:** Mary Kathryn Poole, Angie L. Cradock, Erica L. Kenney

**Affiliations:** 1 Department of Nutrition, Harvard T.H. Chan School of Public Health, Boston, Massachusetts; 2 Department of Social and Behavioral Sciences, Harvard T.H. Chan School of Public Health, Boston, Massachusetts

## Abstract

In 2017, the US Department of Agriculture’s Child and Adult Care Food Program’s (CACFP’s) nutrition standards were updated to improve nutrition and meal quality while remaining feasible for child care providers to implement. We conducted a pre–post study of 13 family child care home (FCCH) providers in Boston, Massachusetts, to compare reported opportunities for training and technical assistance and knowledge of new nutrition standards before the effective date of the updates (October 1, 2017) and 1 year later. The McNemar test was used to test for differences in provider responses. Few FCCH providers received training or technical assistance or had knowledge of most new standards at baseline or at follow-up; however, provider-reported knowledge of the whole-grain standard improved over time (from 6 providers to 12 providers) (*P* = .03). One year post implementation, FCCH providers still needed additional training, technical assistance, or other support to meet the new nutrition standards for meals served to children.

**Summary**

**What is already known on this topic?**

It is unknown whether providers in Boston Family Child Care Homes (FCCHs) are supported in implementing the new Child and Adult Care Food Program’s nutrition standards, which were enacted in 2017.

**What is added by this report?**

Few FCCH providers attended a training or received technical assistance for the nutrition standards before the new standards went into effect; uptake had not increased significantly 1 year later. Although most providers were aware of the revised standards, they had little knowledge of what the standards entailed.

**What are the implications for public health practice?**

Training and technical assistance opportunities that address gaps in knowledge about the new standards may be needed to assist FCCHs with their implementation.

## Introduction

More than 1 million children in the United States attend state-licensed Family Child Care Homes (FCCHs) (1), settings in which providers care for a small group of children in their homes. Because child dietary preferences are established early in life (2), child care settings, including FCCHs, contribute to shaping the diets of young children (3,4). Early exposure to a nutritious diet (5) is a key strategy for preventing children from experiencing diet-related disease and excess weight in adulthood (6,7).

The Child and Adult Food Care Program (CACFP) reimburses participating child care providers who serve low-income children for meals that adhere to a set of nutrition standards. On October 1, 2017, CACFP adopted its updated nutrition standards as required by the Healthy Hunger Free Kids Act of 2010 (8), which incorporated nutrition recommendations from the National Academy of Medicine (9). The updates (Table) were designed to improve nutrition while also being simple and cost-neutral for child care providers.

**Table:** Changes in the Child and Adult Care Food Program’s Nutrition Standards for Children Aged 3 to 5 Years, 2017^a^

Nutrition Standard	Prior to October 1, 2017	October 1, 2017 to Present
Juice	Juice may be served at snack	Limit 100% juice to 1 serving daily
Fruits and vegetables	Serve a vegetable or fruit at lunch	Serve both a fruit and vegetable at lunch
Whole grains	Serve grains that are either whole, enriched, or fortified	Serve whole grains at least once daily
Grain-based desserts	No restrictions for grain-based desserts	Eliminate grain-based desserts
Milk	Serve unflavored or flavored low-fat or fat-free milk	Serve unflavored low-fat or fat-free milk
Frying	No restrictions for on-site frying	Eliminate frying on-site as a cooking method

a US Department of Agriculture, Food and Nutrition Services (10).

Sponsoring organizations that assist CACFP-participating FCCH providers with the reimbursement process are also responsible for helping providers meet CACFP nutrition standards. It is not clear, however, whether FCCH providers, whose expertise is child care, not nutrition, receive training, technical assistance, and other forms of support sufficient for being fully compliant with the revisions. Two studies of child care centers before implementation found that some were already meeting the new standards while others were not (11,12). No studies, however, have explored whether FCCHs are meeting the new standards or whether their staff members receive training, technical assistance, or support to ensure successful adoption. FCCHs are small, may not frequently participate in nutrition trainings (13), and have reported the need for convenient, low-cost trainings (13,14). Understanding FCCH providers’ needs for training, technical assistance, and other supports can help identify possible strategies for supporting complete implementation of CACFP nutrition standards.

## Purpose and Objectives

We studied the experience of Boston FCCH providers with implementing new CACFP standards before the policy effective date and 1 year later. Our first aim was to understand the extent to which FCCH providers had access to training, technical assistance, and other support for the new standards. We hypothesized that few providers would have received support at either point. Our second aim was to understand providers’ awareness and knowledge of what the new standards entailed. We hypothesized that few were aware of the standards at baseline but that knowledge improved by follow-up.

## Intervention Approach

CACFP nutrition standards for preschool-aged children were revised on October 1, 2017, to include 1) serving a fruit and vegetable with lunch, 2) limiting 100% juice to 1 serving per day, 3) serving whole grains for at least 1 grain component, 4) prohibiting reimbursement for grain-based desserts, 5) serving unflavored skim or 1% milk, and 6) removing on-site frying as a cooking method (8).

## Evaluation Methods

### Study design and population

Our pre–post study was led by a team of Boston-area researchers. Baseline data were collected from August 1 through October 1, 2017 (the effective date for CACFP revisions), and follow-up data were collected approximately 1 year later, from July through December 2018. Eligible participants were licensed FCCH providers in Boston, Massachusetts, who participated in CACFP and served at least 1 child aged 3 to 5 years; we had no other exclusion criteria. Providers were identified from a publicly available list of Boston’s 396 FCCHs with a goal of recruiting 30 providers at baseline. Researchers selected and recruited the 30 providers from the list by using a computer-based random number generator. Providers were notified of the study through a flyer. Researchers then contacted providers by telephone, and by email when available, to verify eligibility criteria and to recruit participants. Providers who did not respond after 3 attempts were considered nonrespondents. Study participants received a $40 gift card following data collection. The same group of participants were recontacted 1 year later and recruited to participate in follow up by using the same recruitment methods, eligibility criteria, and incentive. This study was classified as nonhuman subjects research by Harvard T.H. Chan School of Public Health, Office of Human Research Administration and exempt from institutional board review.

### Measures

No validated survey tools were available for our study objectives, so we designed a survey to assess whether FCCH providers were prepared for new CACFP nutrition standards and had received support in implementing them. We obtained input from a local CACFP sponsor and city health officials who worked with FCCH providers on nutrition to develop close-ended survey questions capturing 2 outcomes: 1) uptake of training and other supports for adopting the nutrition standards and 2) understanding of the nutrition standards. Researchers distributed the 20-minute survey, available in English and Spanish, to FCCH providers at baseline and at 1-year follow-up. On the survey, FCCH providers reported their primary language (English or Spanish), years of program operation, and number of enrolled children aged 3 to 5 years. They reported whether they were aware of any trainings on the new CACFP standards, had attended a training, and had any barriers to attendance. They also indicated whether they had received technical assistance with developing menus and rated their interest in implementation support, including trainings, sample menus, coaching from nutrition professionals, guidance on food costs, criteria for whole grains, and grocery lists.

FCCH providers’ awareness of CACFP standards was measured with a survey question asking if providers had heard about the new standards. Knowledge of standards was assessed with multiple-choice questions prompting providers to select the updated standards for juice, milk, fruits and vegetables, whole grains, grain-based desserts, and on-site frying.

### Analysis

We used descriptive statistics to summarize FCCH characteristics, uptake of training and technical assistance, interest in support, and understanding of CACFP standards. We used the McNemar test to test for differences in binary outcomes between baseline and follow-up measures by using SAS University Edition, version 3.8 (SAS Institute, Inc). Significance was set at *P* < .05.

## Results

Of the 263 FCCHs we initially contacted, 56 (21%) were ineligible (19 did not participate in CACFP and 37 did not care for children aged 3 to 5 years). One hundred twenty-four providers (47%) declined, citing lack of time or interest. Ninety-seven (37%) were nonresponders, and 29 (11%) agreed to participate in the study. At 1-year follow-up, 13 FCCHs with baseline and follow-up measures remained. Of the 16 providers lost to follow-up, 4 were no longer in operation, 4 no longer enrolled children aged 3 to 5 years, and 8 were too busy to participate. Providers lost to follow-up were more likely to speak Spanish, but their programs did not differ by size.

**FCCH characteristics.** Providers served an average of 3 (standard deviation [SD], 2) children aged 3 to 5 years at baseline and 4 (SD, 2) at follow-up. FCCHs had been in operation for 4 (SD, 1) years on average. Most reported speaking English as their primary language. FCCHs in our sample did not differ significantly by neighborhood or primary language spoken from nonparticipating Boston FCCHs.

**Training, technical assistance, and support.** At baseline, 8 of the 13 participating FCCH providers indicated an interest in training on the new standards, 7 were aware of such a training, and 5 had completed one. Inconvenient time was the most common barrier, cited by 3 providers. By follow-up, 9 providers were aware of a training, 8 had attended a training, and 3 were interested in training. At baseline, 4 reported receiving technical assistance with menus compared with 5 by follow-up.

Participant responses indicating agreement about whether certain supports were helpful changed from baseline to follow-up as follows: grocery lists, 10 at baseline and 8 at follow-up; sample menus, 7 at baseline and 5 at follow-up; criteria for whole grains, 7 at baseline and 6 at follow-up; guidance on costs: 5 at baseline and 5 at follow-up. No significant changes in requests for training and support were found.

**Understanding of standards.** At baseline, providers were unaware of the CACFP revisions; however, all providers were aware by follow-up. Providers identified an average of 2 of the 6 standards at baseline and 3 at follow-up. Though all providers identified at least 1 standard at follow-up, their knowledge of most standards remained incomplete. Providers were most familiar with the standard for whole grains, and this proportion was significantly higher at follow-up (6 at baseline and 12 at follow-up; *P* = .03). No other changes in knowledge of standards reached significance. Fewer than half of providers correctly identified the new standards for milk, fruits and vegetables, and on-site frying. Knowledge remained lowest for the grain-based dessert standard with only 3 providers correctly identifying the new standard at follow-up.

## Implications for Public Health

In fall 2017, most Boston FCCH providers in our study sample were aware of the upcoming CACFP revisions; however, at baseline few said they had attended a training on the standards. At 1-year follow-up providers indicated an interest in support for implementing the new standards, such as training on whole grain criteria, grocery lists, and sample menus; however, knowledge of most CACFP standards was low at both points. Although few FCCH providers reported at baseline that they had attended a training on CACFP nutrition standards, this number increased by follow-up, mirroring the finding from Tovar et al that providers attended up to 1 training per year (13). Nevertheless, at baseline only 4 providers had received technical assistance and only 5 by follow-up. Our results suggest that training alone may not be sufficient. Although many providers had attended 1 training by follow-up, their knowledge of most of the standards remained low. Time of day was noted as a barrier to training. Convenient training opportunities such as web-based trainings or technical assistance visits that focus on grocery lists, menus, and whole grain criteria could be areas of focus for supporting adoption of CACFP standards.

At baseline and follow-up, knowledge of new CACFP standards was low. Our results differed from Chriqui et al who found that most child care center providers were aware of the new CACFP standards before their implementation (12). Although providers in our study sample were aware that standards had changed, many were unable to identify the new standards. This finding may be a result of our using a multiple-choice response format rather than reporting general awareness. It is also possible that our findings indicate that FCCHs have different implementation challenges than child care centers. Gaps in knowledge suggest potential priority areas for support by highlighting which standards were less well-known to providers.

Our study is the first, to our knowledge, to document changes in both provider knowledge of new CACFP nutrition standards and support received before implementation of those standards and 1 year following. Our findings illustrate the importance of ensuring that training, technical assistance, and other support are made available to and accessed by FCCHs to ensure full understanding and implementation of nutrition standards. However, our study had several limitations. We included only a small number of Boston FCCHs; therefore, our findings may not be generalizable. Additionally, more than half of the initially participating providers were lost to follow-up, and more than half of these were Spanish-speaking providers, which limits our sample’s representativeness of Boston FCCHs. 

Future studies may expand on our findings by recruiting a larger sample of English- and Spanish-speaking FCCHs from multiple locales and by oversampling to account for losses to follow-up or eligibility changes. Such studies may assist FCCH providers with training, technical assistance, or other supports to further their implementation of new nutrition standards for meals served to children.
